# Injectable Estradiol Dosing Regimens in Transgender and Nonbinary Adults Listed as Male at Birth

**DOI:** 10.1210/jendso/bvaf004

**Published:** 2025-01-21

**Authors:** Maralee Kanin, Margaret Slack, Reema Patel, Kuan-Ting Chen, Nicholas Jackson, Kristen C Williams, Shira Grock

**Affiliations:** Division of Endocrinology, Diabetes & Metabolism, University of California, Los Angeles, 10833 Le Conte Ave, 57-145 CHS, Los Angeles, CA 90095, USA; Department of Medicine, Internal Medicine Residency, University of California, Los Angeles, 757 Westwood Plaza, Suite #7236, Los Angeles, CA 90095, USA; Division of Endocrinology, Diabetes, and Metabolism, Department of Medicine, VA Greater Los Angeles Healthcare System, Los Angeles, 11301 Wilshire Boulevard, Los Angeles, CA 90073, USA; Department of Medicine Statistics Core, David Geffen School of Medicine, University of California, Los Angeles, 911 Broxton Ave, 3rd floor, Los Angeles, CA 90095, USA; Department of Medicine Statistics Core, David Geffen School of Medicine, University of California, Los Angeles, 911 Broxton Ave, 3rd floor, Los Angeles, CA 90095, USA; Department of Urology, David Geffen School of Medicine, University of California, Los Angeles, 10833 Le Conte Avenue Box 951738, Los Angeles, CA 90095, USA; Division of Endocrinology, Diabetes & Metabolism, University of California, Los Angeles, 10833 Le Conte Ave, 57-145 CHS, Los Angeles, CA 90095, USA

**Keywords:** transgender women, injectable estradiol, estradiol ranges

## Abstract

**Context:**

Many transgender and nonbinary (TGNB) individuals assigned male at birth (AMAB) seek hormone therapy to achieve physical and emotional changes. Standard therapy includes estradiol, with or without an antiandrogen. Our clinical observations suggest that currently recommended injectable estradiol dosing may lead to supratherapeutic estradiol levels.

**Objective:**

We sought to evaluate whether lower-than-recommended doses of injectable estradiol were effective in achieving serum estradiol and testosterone goals.

**Methods:**

We conducted a retrospective cohort study to evaluate injectable estradiol dosing in treatment-naive AMAB individuals initiating hormone therapy. Data from a single provider at an academic center from January 2017 to March 2023 were analyzed. A total of 29 patients were eligible for inclusion. The primary variables of estradiol dosage, serum estradiol, and testosterone levels were analyzed over 15 months.

**Results:**

The average estradiol dose decreased from 4.3 to 3.7 mg weekly (*P* < .001) during the study period with a final on-treatment estradiol level of 248 pg/mL. All individuals achieved a testosterone level of less than 50 ng/dL during the study period. The average initial on-treatment testosterone level was not significantly different from average final on-treatment measurement of 24.0 mg/dL (*P* = .95). Spironolactone use at study initiation was not associated with a lower initial on-treatment testosterone level, though it was associated with a lower estradiol level of 285 pg/dL compared to 427 pg/dL for those on estradiol monotherapy (*P* = .017).

**Conclusion:**

Lower doses of injectable estradiol can achieve therapeutic estradiol levels with excellent testosterone suppression. Spironolactone was not associated with additional testosterone suppression and may result in lower estradiol levels.

Many transgender and nonbinary (TGNB) individuals seek gender-affirming hormone therapy (GAHT) to achieve physical and emotional changes that better represent their gender identity [1, 2]. For TGNB individuals assigned male at birth (AMAB), GAHT typically includes estradiol, with or without an antiandrogen. While dependent on patient-specific goals, estradiol is often titrated to achieve hormone concentrations equivalent to those of reproductive-aged cis women: an estradiol level of 100 to 200 pg/mL and a testosterone level less than 50 ng/dL [3, 4]. However, limited data with relatively weak justifications support the currently recommended regimens.

There is inconsistency in estradiol dosing recommendations between different clinical practice guidelines. The 2017 Endocrine Society clinical practice guideline for the endocrine treatment of gender-dysphoric/gender-incongruent individuals recommends estradiol valerate or cypionate parenterally 2 to 10 mg weekly [3]. The University of California, San Francisco (UCSF) guidelines for the primary and gender-affirming care of TGNB people recommend initiating estradiol valerate at 3 to 5 mg parenterally weekly with a maximum dose of 20 mg weekly or estradiol cypionate 1 to 2 mg weekly with a maximum dose of 5 mg weekly [5]. While there is some overlap in recommended doses, UCSF recommends estradiol valerate doses that are up to double the recommended dose in the Endocrine Society guidelines. Furthermore, UCSF recommends a significantly lower dose of estradiol cypionate, while the Endocrine Society guidelines do not differentiate between estradiol valerate and estradiol cypionate. While the Endocrine Society guidelines were developed with more rigorous methodology compared to the USCF guidelines, both guidelines are widely referenced in clinical practice and discrepancies should be studied further.

The study teams’ clinical observations, as well as findings published in recent literature, led us to hypothesize that current guideline-based dosing of injectable estradiol can result in supratherapeutic estradiol levels [6-8]. In this study, we aimed to evaluate the effect of injectable estradiol dose on serum estradiol and testosterone concentrations in TGNB adults AMAB.

## Materials and Methods

A retrospective case series of TGNB individuals AMAB newly prescribed injectable estradiol by a single provider at an academic referral center from January 2017 to March 2023 was performed. The study period coincides with the formation of the University of California, Los Angeles, Gender Health Program. This study was approved by the UCLA Institutional Review Board.

Eligible patients were AMAB individuals aged 18 to 89, who were GAHT-naive and prescribed injectable estradiol. Participants were excluded if they had a history of orchiectomy, current use of gonadotropin-releasing hormone agonists, or had been on estradiol-containing medications within 6 months prior to starting injectable estradiol.

Patients were identified using structured query language to extract data from our institution's electronic health record system. All patients prescribed injectable estradiol during the study period were identified, and patient records were reviewed by at least 2 team members for eligibility. Two study team members abstracted data using electronic case report forms built in REDCap with data validation rules to minimize potential human error. Manual chart review was used to extract demographic information, estradiol dose, serum hormone levels, use of antiandrogens, and use of progesterone during the 15-month study period. For each case, all visits with the treating provider and laboratory values were assessed from time of first visit with a provider until 15 months after the initial estradiol prescription, anticipating that it would take up to 12 to 15 months to establish a stable hormone regimen. A second abstractor confirmed the accuracy of the data by reviewing laboratory values outside anticipated clinical ranges.

Estradiol and testosterone levels were measured at UCLA, Labcorp, Quest Diagnostics, and ARUP Laboratories. UCLA uses electrochemiluminescence testing with the option for send-out to ARUP if measurement via liquid chromatography–mass spectrometry (LC-MS) is desired. Quest Diagnostics completes measurements via immunoassay or LC-MS depending on the test ordered and LabCorp similarly offers electrochemiluminescence or LC-MS. All patients were prescribed once-weekly subcutaneous (SQ) (n = 28) or intramuscular (IM) (n = 1) estradiol. Patients were instructed by a single provider to check laboratory test results midway through their injection cycle, though this could not be verified given the retrospective nature of the study.

### Statistical Analysis

Primary outcomes included serum estradiol levels, serum testosterone levels, and the percentage of patients achieving target hormone levels (defined as testosterone <50 ng/dL and estradiol 100-200 pg/mL). To examine changes over time in serum estradiol, serum testosterone, and estradiol dose, we used mixed-effects linear regression models with time period fixed effects and random intercept for patients with nested random slope for time period. Time periods were defined as 1) prior to treatment (pre-treatment), 2) the first measurement occurring at least 30 days after treatment (initial on-treatment), and 3) the last measurement available while on treatment during the study period (final on-treatment). Mixed-effects linear regression models were also used to assess associations of estradiol dose with initial on-treatment hormone levels. Similar linear and logistic mixed-effects modeling was performed to compare mean and target (<50 ng/dL) testosterone measurements between different serum estradiol groups (<100; 100-200; >200). Last, subsample analyses among those initiated on 4 to 5 mg of estradiol were examined for change in pre-treatment to initial on-treatment hormone measurements as a function of spironolactone use. Analyses were conducted in Python version 3.9 and Stata MP version 18.

## Results

**Figure 1.**
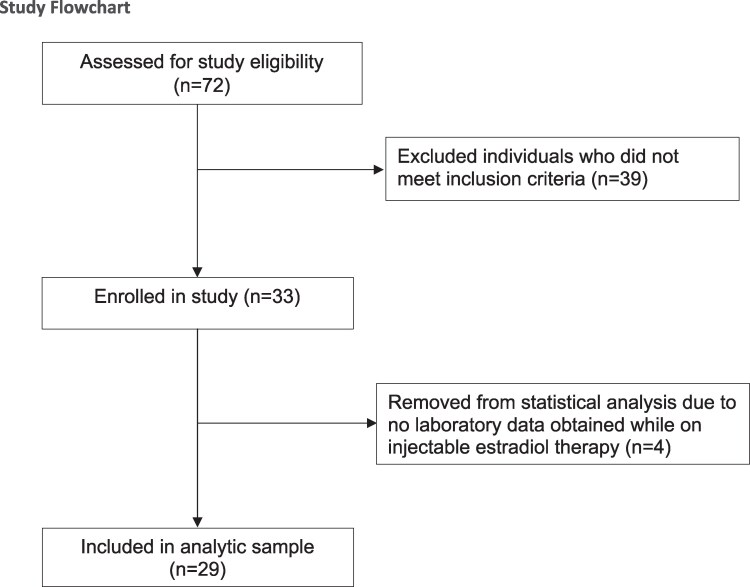
Study flowchart. Flowchart of study design and patient disposition.

Our initial query identified 74 individuals prescribed injectable estradiol by the relevant provider. Of these, 33 patients were eligible for inclusion (Fig. 1). Four patients were subsequently excluded from statistical analysis due to lack of laboratory data following initiation of treatment. The final analytic sample was 29 patients. After pretreatment and initial on-treatment levels were obtained, 4 participants exited the study prematurely due to orchiectomy (n = 2) or switching route of estradiol administration to a noninjectable form (n = 2).

Baseline patient characteristics are listed in Table 1. The average age of patients at initiation of estrogen therapy was 29.4 years. A total of 31% of study participants identified as White, 10% identified as Asian or Pacific Islander, and 62% opted not to respond or marked unknown.

**Table 1.** Participant demographics

	Values
Age at first Tx, mean (SD), y	29.4 (6.7)
*^a^* BMI, mean (SD)	26.6 (6.3)
*^b^* Race, (N) %	
African American or Black	(0) 0%
Alaska Native/American Indian	(0) 0%
Asian or Pacific Islander	(3) 10%
White, European, or Middle Eastern	(9) 31%
Unknown/No response	(18) 62%
Ethnicity, (N) %	
Hispanic	(1) 3%
Not Hispanic	(10) 34%
Unknown/No response	(18) 62%
Method of delivery, (N) %	
Intramuscular	(1) 3%
Subcutaneous	(28) 97%

Patient demographics are reported.

Abbreviations: BMI, body mass index; Tx, treatment.

^*a*^BMI within 90 days prior to treatment; N = 25.

^*b*^Categories are not mutually exclusive.

Starting doses of injectable estradiol ranged from 2.5 to 10.0 mg/week. The majority of study participants (27/29) were started on a weekly dose between 3 and 5 mg, with an average starting dose of 4.3 mg (SD = 1.3). Only 1 participant was treated with estradiol cypionate; 28 participants were prescribed estradiol valerate.

### Estradiol

The initial on-treatment laboratory test results were obtained an average of 85 days after estradiol initiation. The average initial on-treatment estradiol level was 347 pg/mL (SD = 177) (Table 2). Only 6 of 29 participants (21%) achieved goal initial on-treatment estradiol levels between 100 and 200 pg/mL. Of the 23 patients who did not achieve goal estradiol levels, 22 (95.6%) were supratherapeutic, with estradiol levels over 200 pg/mL. Seventeen of 29 participants (59%) achieved at least 1 estradiol level in the 100 to 200 pg/mL range during the study period (Table 3). The average estradiol dose prescribed to patients at the time points when they achieved a goal estradiol level was 3.4 mg weekly (range, 2.5-5 mg), whereas the average estradiol dose for participants with estradiol levels greater than 200 pg/mL was 4.2 mg weekly (*P* = .028) (Table 4). The single patient on estradiol cypionate was initiated at 2.5 mg weekly and initial on-treatment estradiol level was 257 mg/dL. The patient remained on 2.5 mg weekly and the second on-treatment estradiol level was 119 mg/dL, after which time the patient switched to estradiol valerate due to limited availability of estradiol cypionate.

**Table 2.** Change in hormones and hormone therapy

	Measurement prior to Tx mean (SD)	First measurement 30+ d post Tx mean (SD)	Final measurement on Tx mean (SD)	First measurement vs pre-Tx *P^a^*	Final measurement vs pre-Tx *P^a^*	Final measurement vs first *P^a^*
Testosterone*^b^*	494 (191)	25.8 (28.6)	24.0 (23.5)	<.001	<.001	.95
Estradiol*^c^*	22.4. (8.5)	347 (177)	248 (115)	<.001	<.001	.005
Estrogen*^d^*	—	4.3 (1.3)	3.7 (1.4)			<.001

Serum testosterone and estradiol levels, along with estradiol dosages during three specified time periods. Time periods were defined as 1) prior to treatment (pre-tx), 2) first measurement occurring at least 30 days after treatment (initial on-tx), and 3) last measurement available while on treatment during the study period (final on-tx).

Abbreviation: Tx, treatment.

^*a*^Mixed-effects linear regression with patient random intercept.

^*b*^Ns (26, 27, 25).

^*c*^Ns (18, 27, 25).

^*d*^Ns (29, 27).

**Table 3.** Overview of treatment outcomes

	Values
Testosterone ever reached <50, (N) %	
No	(0) 0%
Yes	(29) 100%
Testosterone < 50 on first lab check after treatment initiation, (N) %	
No	(3) 10%
Yes	(26) 90%
Estradiol ever 100-250, (N) %	
No	(4) 14%
Yes	(25) 86%
Estradiol 100-250 on first lab check after treatment initiation, (N) %	
No	(21) 72%
Yes	(8) 28%
Changed estrogen administration route, (N) %	
No	(26) 90%
Yes	(3) 10%

Treatment outcomes for hormone levels and changes in route of administration.

**Table 4.** Differences in testosterone by estradiol group

	Group 1 estradiol <100 (N = 6)	Group 2 estradiol 100-200 (N = 23)	Group 3 estradiol >200 (N = 71)	Group 1 vs 2 *P*	Group 1 vs 3 *P*	Group 2 vs 3 *P*
Testosterone						
Mean	46.1	23.2	21.6	.012*^a^*	.006*^a^*	.75*^a^*
% < 50 ng/dL	86.3%	89.5%	91.9%	.77*^b^*	.57*^b^*	.71*^b^*

Differences in testosterone levels based on estradiol group.

^*a*^Mixed-effects linear regression with patient random intercept.

^*b*^Mixed-effects logistic regression with patient random intercept.

**Figure 2.**
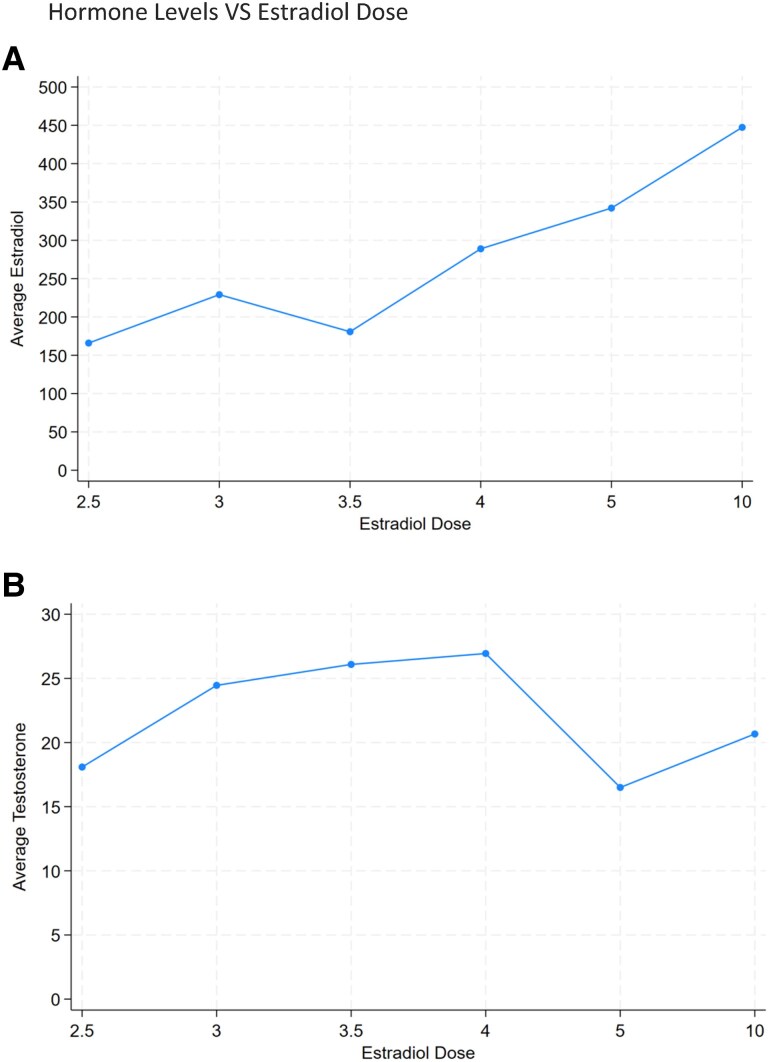
Hormone levels vs estradiol dose. Composite A, estradiol levels and B, testosterone levels plotted relative to the estradiol dosage for all labs more than 30 days after treatment initiation.

Estradiol levels were measured periodically throughout the study and are plotted relative to the prescribed estradiol dose in Fig. 2A. Using the composite data, each 1-mg increase in estradiol dose corresponded to an average increase in serum estradiol by 42.6 pg/dL (*P* < .001). Over the course of the 15-month study period, the average prescribed estradiol dose decreased by 0.6 mg from 4.3 to 3.7 mg weekly (*P* < .001). The mean final on-treatment estradiol level was 248 pg/mL (SD = 115), which was significantly lower than the mean initial on-treatment measurement (*P* < .005), as denoted in Table 2.

### Testosterone

Testosterone levels were obtained in most individuals prior to treatment initiation. The average pretreatment testosterone level was 494 ng/dL with an SD of 191 mg/dL. The initial on-treatment testosterone level decreased to an average of 25.8 (*P* < .001). A total of 90% of individuals achieved the target testosterone level of less than 50 ng/dL on their initial on-treatment laboratory draw (see Table 3), which was obtained an average of 85 days after initiation of estradiol. Testosterone levels were monitored throughout the study period and are seen plotted relative to estradiol dose in Fig. 2B. Over the course of the study period, all participants obtained at least 1 goal testosterone level less than 50 ng/dL. The mean final on-treatment serum testosterone was 24.0 ng/dL, which was significantly lower than pretreatment levels (*P* < .001) but did not differ significantly from mean initial on-treatment values (*P* = .95). At time points when patients achieved an estradiol level of 100 to 200 pg/dL the mean testosterone level was 23.2 ng/dL. Mean testosterone at time points when patients had an estradiol level greater than 200 pg/dL was similar at 21.6 ng/dL (*P* = .75), while mean serum testosterone at time points when estradiol was less than 100 pg/dL was higher at 46.1 ng/dL (*P* = .01) (see Table 4). There were no statistically significant changes in testosterone levels between the various prescribed estradiol doses (*P* = .72).

### Antiandrogens

**Figure 3.**
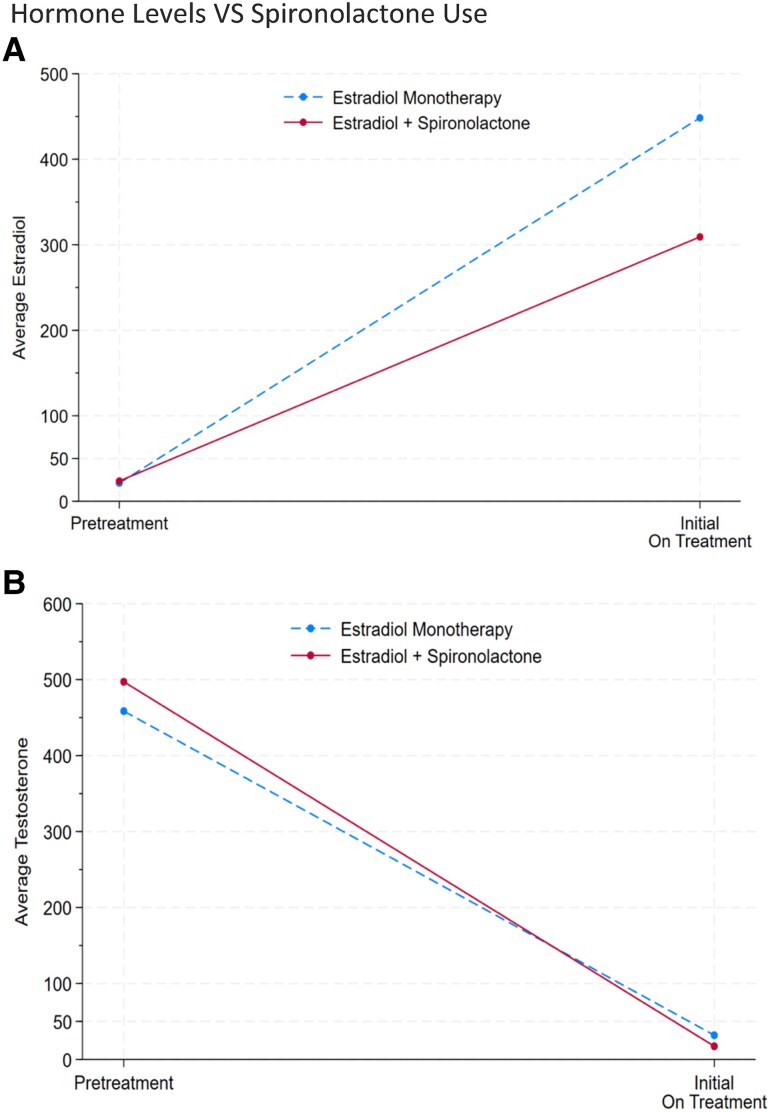
Hormone levels vs spironolactone use. Subgroup analysis performed for patients on estradiol 4 to 5 mg weekly at initiation of therapy. Analysis compared change in A, serum estradiol and B, testosterone from pretreatment to initial on-treatment levels for individuals on estradiol monotherapy compared to estradiol plus spironolactone.

Subgroup analysis was performed for patients on estradiol 4 to 5 mg weekly at study initiation. Change in hormone levels from pretreatment to initial on-treatment measurements were compared between the estradiol-only group and individuals initiated on combination therapy with estradiol and spironolactone. There was no reliable difference in the change in testosterone level for those using spironolactone (n = 9) compared to those on estradiol monotherapy (n = 12) (difference-in-difference mean = −53; *P* = .49; Fig. 3B). However, the initial on-treatment estradiol level differed significantly between groups. As seen in Fig. 3A, patients on combination therapy (n = 9) achieved a mean initial on-treatment estradiol level of 285 pg/dL compared to 427 pg/dL for those on estradiol monotherapy (n = 9). The change in serum estradiol from pretreatment to initial on-treatment levels was statistically different between groups (difference-in-difference mean = −142; *P* = .017). The average weekly estradiol dose was 4.3 mg for estradiol monotherapy and 4.5 mg for combination therapy (*P* = .37). The dose of spironolactone was either 50 mg daily (n = 6) or 100 mg daily (n = 3).

Antiandrogen use apart from spironolactone included 1 patient who was prescribed bicalutamide 18 days after estradiol initiation and 3 patients prescribed finasteride between 176 and 405 days after starting estrogen. A total of 10 individuals were prescribed progesterone on patient request. All prescriptions occurred more than 258 days after estradiol initiation. Of the patients on progesterone, 5 were prescribed 100 mg daily and 5 were prescribed 200 mg daily.

## Discussion

Our study demonstrated that lower doses of injectable estradiol can be used to achieve therapeutic estradiol levels with a high rate of testosterone suppression. Initiating lower-dose injectable estradiol can avoid supratherapeutic serum levels and may reduce side effects and associated risks of estradiol therapy such as venous thromboembolism [9].

While prior studies have similarly concluded that guideline-based injectable estradiol regimens may lead to supratherapeutic levels [7, 8], this is the first study, to our knowledge, to evaluate the use of injectable estradiol in GAHT-naive individuals. This approach allowed for evaluation of additional outcomes including time to testosterone suppression and rate of discontinuing injectable estradiol.

Similar to our study, Herndon and colleagues [7] observed that gender-diverse individuals on an average of 3.75 mg weekly subcutaneous estrogen had an average mid-cycle estradiol level of 196 pg/mL. On the contrary, other studies suggest higher doses of injectable estradiol may also result in target hormone levels; however, these studies mostly assessed estradiol trough levels instead of mid-cycle levels [8, 10].

Based on our data, an average estradiol dose of 3.7 mg weekly led to supratherapeutic levels. Thus, it may be prudent to initiate injectable estradiol at a dosage closer to 3 mg weekly. While only one patient in our study was on estradiol cypionate, the patient achieved a goal estradiol level on 2.5 mg weekly, suggesting that the ideal dosing for estradiol cypionate and estradiol valerate may be similar. We observed that every 1-mg increase in estradiol dose resulted in an average increase in serum estradiol by 42.6 pg/dL (*P* < .001). These data can be a starting point for clinical dose adjustments. When adjusting for supratherapeutic levels, our data reassuringly suggest that injectable estradiol can safely be lowered, at least to a moderate extent, without a large increase in serum testosterone. In our study, even individuals with estradiol less than 100 pg/mL had a testosterone level at goal 86.3% of the time.

Of the 29 patients evaluated, only 2 patients (6.8%) switched from injectable to another form of estradiol. Our data offer preliminary evidence that patients are sufficiently satisfied with injectable estradiol (particularly SQ injections) to maintain its use for at least the first 15 months of treatment.

For individuals initiated on 4 to 5 mg injectable estradiol, use of spironolactone was not associated with lower testosterone levels and most individuals on injectable estradiol achieved rapid testosterone suppression without antiandrogens. While spironolactone was not associated with changes in serum testosterone, we did observe a significantly lower estradiol level in patients initiated on combination therapy with spironolactone compared to those on estradiol monotherapy. This was also observed by Leinung et al [11] in a study of gender-diverse individuals using oral estradiol. The clinical significance of this finding is unclear, as estradiol levels were still within goal and physical changes were not measured. Additionally, we cannot rule out the possibility that higher doses of spironolactone or lower initial on-treatment serum estradiol levels could result in different findings.

While use of progesterone in GAHT remains highly controversial, patients often request progesterone for anecdotal improvement in breast growth and libido [12]. While progesterone may have an antiandrogenic effect [12], all patients in this study achieved a suppressed testosterone prior to initiation of progesterone and were on progesterone only toward the final months of the study period. In this context, it is unlikely that progesterone significantly affected the results. Regardless, additional studies are needed to assess the risks and benefits of progesterone and its effect on testosterone suppression.

Our study is characterized by typical limitations and strengths of case series. The study sample was limited to a single provider at an academic center serving a primarily White and privately insured population [13]. Although the provider consistently instructed patients on accurate dosing and mid-cycle timing for blood draws, we could not control for individual variance in dosing schedule, route of administration (SC vs IM), or timing of blood draws. Most laboratory values were assessed at the clinical laboratory for UCLA or by a common commercial laboratory group, but variance in laboratory standards and use of assays outside LC-MS may have introduced bias or error.

## Conclusion

Our study demonstrated that lower doses of injectable estradiol can achieve hormone levels in the therapeutic range for gender-diverse individuals. It is not clear that spironolactone results in lower testosterone levels when used with injectable estradiol. Combination therapy with spironolactone and injectable estradiol may be associated with lower serum estradiol levels compared to estradiol monotherapy. Based on this study, along with other published literature, the upper limit of the recommended dose range for injectable estradiol may need to be lowered. Further data are needed to address some of the uncertainties surrounding the use of spironolactone.

## Disclosures

The authors do not have any disclosures or conflicts of interest to declare.

## Data Availability

Original data generated and analyzed during this study are included in this published article or in the data repositories listed in “References.”

## Abbreviations

AMAB: assigned male at birth
GAHT: gender-affirming hormone therapy
IM: intramuscular
LC-MS: liquid chromatography–mass spectrometry
SQ: subcutaneous
TGNB: transgender and nonbinary
UCLA: University of California, Los Angeles
UCSF: University of California, San Francisco
